# Mutations in *N*-acetylglucosamine (*O*-GlcNAc) transferase in patients with X-linked intellectual disability

**DOI:** 10.1074/jbc.M117.790097

**Published:** 2017-06-05

**Authors:** Anke P. Willems, Mehmet Gundogdu, Marlies J. E. Kempers, Jacques C. Giltay, Rolph Pfundt, Martin Elferink, Bettina F. Loza, Joris Fuijkschot, Andrew T. Ferenbach, Koen L. I. van Gassen, Daan M. F. van Aalten, Dirk J. Lefeber

**Affiliations:** From the ‡ Department of Neurology, Donders Institute for Brain, Cognition and Behaviour and; the ‖ Department of Genetics, Radboud University Medical Centre, 6500 HB Nijmegen, The Netherlands,; the § Department of Laboratory Medicine, Translational Metabolic Laboratory, Radboud Institute for Molecular Life Sciences, Radboud University Medical Center, 6525 GA Nijmegen, The Netherlands,; the ¶ Centre for Gene Regulation and Expression, School of Life Sciences, University of Dundee, DD1 5EH Dundee, Scotland, United Kingdom,; the ** Department of Genetics, University Medical Centre Utrecht, 3508 AB Utrecht, The Netherlands,; the ‡‡ Department of Paediatrics, VieCuri Hospital, 5900 BX Venlo, The Netherlands, and; the §§ Department of Paediatrics, Radboud University Medical Centre and Amalia Children's Hospital, 6500 HB Nijmegen, The Netherlands

**Keywords:** glycobiology, glycosyltransferase, metabolic disease, O-GlcNAcylation, O-linked N-acetylglucosamine (O-GlcNAc) transferase (OGT), Congenital Disorders of Glycosylation, Host Cell Factor 1 (HCF-1), X-linked Intellectual Disability

## Abstract

*N*-Acetylglucosamine (*O*-GlcNAc) transferase (OGT) regulates protein *O*-GlcNAcylation, an essential and dynamic post-translational modification. The *O*-GlcNAc modification is present on numerous nuclear and cytosolic proteins and has been implicated in essential cellular functions such as signaling and gene expression. Accordingly, altered levels of protein *O*-GlcNAcylation have been associated with developmental defects and neurodegeneration. However, mutations in the *OGT* gene have not yet been functionally confirmed in humans. Here, we report on two hemizygous mutations in *OGT* in individuals with X-linked intellectual disability (XLID) and dysmorphic features: one missense mutation (p.Arg284Pro) and one mutation leading to a splicing defect (c.463–6T>G). Both mutations reside in the tetratricopeptide repeats of OGT that are essential for substrate recognition. We observed slightly reduced levels of OGT protein and reduced levels of its opposing enzyme *O*-GlcNAcase in both patient-derived fibroblasts, but global *O*-GlcNAc levels appeared to be unaffected. Our data suggest that mutant cells attempt to maintain global *O*-GlcNAcylation by down-regulating *O*-GlcNAcase expression. We also found that the c.463–6T>G mutation leads to aberrant mRNA splicing, but no stable truncated protein was detected in the corresponding patient-derived fibroblasts. Recombinant OGT bearing the p.Arg284Pro mutation was prone to unfolding and exhibited reduced glycosylation activity against a complex array of glycosylation substrates and proteolytic processing of the transcription factor host cell factor 1, which is also encoded by an XLID-associated gene. We conclude that defects in *O*-GlcNAc homeostasis and host cell factor 1 proteolysis may play roles in mediation of XLID in individuals with *OGT* mutations.

## Introduction

Protein *O*-GlcNAcylation is a dynamic and reversible post-translational modification that is present on many nucleocytoplasmic and mitochondrial proteins (1, 2). *O*-GlcNAcylation involves cycling of a single *N*-acetylglucosamine (*O*-GlcNAc) ^5^ moiety on serine and threonine residues of a spectrum of substrates, affecting their stability, activity, and subcellular localization (3, 4). The transfer of *O*-GlcNAc is catalyzed by *O*-GlcNAc transferase (OGT) (5, 6) and is modulated by the availability of the donor substrate UDP-GlcNAc (7). UDP-GlcNAc biosynthesis is coupled to glucose, glutamine, and nucleotide metabolism. Concomitantly, OGT exerts a nutrient-sensitive regulation over various cellular functions by acting on myriad targets (8). OGT is highly conserved in metazoa and consists of multiple tetratricopeptide repeats (TPRs) on the N terminus and a C-terminal glycosyltransferase domain (9, 10). Although TPRs are known to be involved in protein-protein interactions, in the context of OGT they facilitate substrate recognition and specificity (11). In addition to its glycosyltransferase activity, OGT was recently shown to have a role in proteolytic processing of host cell factor 1 (HCF1) (12, 13), a transcriptional co-regulator of the cell cycle (14). HCF1 is heavily *O*-GlcNAcylated (15), but a recent study revealed that *O*-GlcNAcylation and proteolytic cleavage of HCF1 by OGT are independent processes (16). OGT activity is counteracted by *O*-GlcNAcase (OGA), a hexosaminidase that specifically removes *O*-GlcNAc modifications (17). It has been shown that chemical inhibition of OGA in different human cell lines leads to a decreased expression of OGT and an increased expression of OGA (18), which indicates an as yet unidentified mechanism of regulation to maintain global *O*-GlcNAc homeostasis. OGT has been linked to embryonic development in a number of animal models. *Caenorhabditis elegans* with null mutations in *ogt-1* are viable and fertile, but with metabolic defects (19). In *Drosophila*, OGT is encoded by the *super sex combs* gene, which is essential for *Drosophila* viability and segmentation (20, 21). Knockdown of *OGT* in zebrafish causes impaired embryonic growth with reduced brain size (22). Although OGT is ubiquitously expressed, it is remarkably abundant in brain (9, 10). Accordingly, *O*-GlcNAcylation has been linked to neuronal function and brain development in a number of cell and animal models (22, 23). In addition, recent studies have shown that *O*-GlcNAc signaling can regulate axonal and dendritic growth (24, 25) and AMPA receptor trafficking (26, 27), possibly affecting learning and memory processes. Also, alterations in protein *O*-GlcNAcylation have been associated with neurodegenerative diseases (28).

Genetic *OGT* variants identified in patients with X-linked intellectual disability (XLID) have previously been documented: first, in a clinical report, albeit accompanied by mutations in *MED12* (a known XLID gene) (29), and second, in a large screen for novel XLID genes (30). Here, we describe two patients with intellectual disability, bearing hemizygous mutations in *OGT*. By combining characterization of the *O*-GlcNAcylation machinery in patient cells and characterization of the effects of OGT XLID mutations on OGT dual activity *in vitro*, we provide preliminary evidence suggesting that effects on the *O*-GlcNAc proteome and proteolytic processing of HCF1 may underpin the observed XLID phenotypes.

## Results

### Clinical phenotype of two ID patients

**Figure 1.**
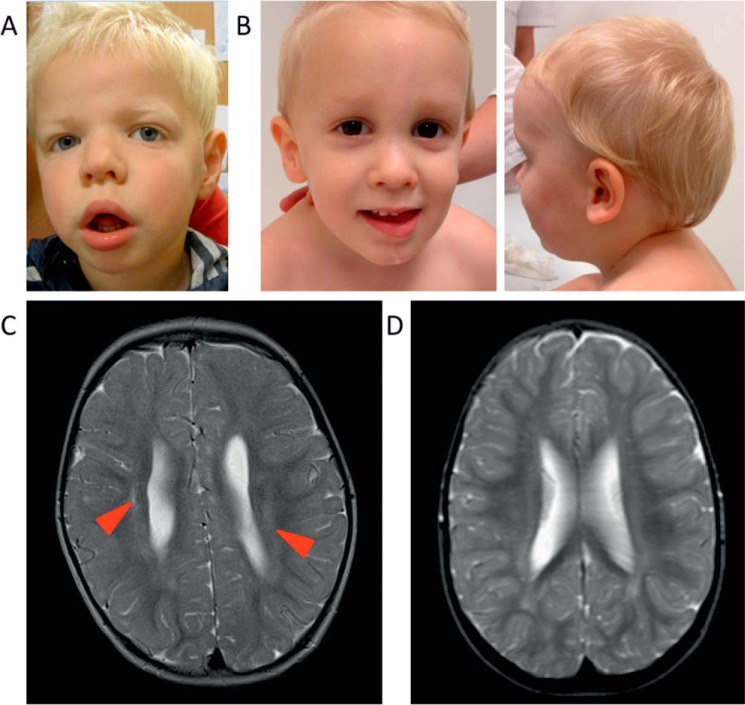
**Clinical images from patients with hemizygous OGT mutations.** *A*, picture of patient 1, showing dysmorphic features. *B*, pictures of patient 2, showing dysmorphic features. *C*, MRI image of patient 1, showing mild abnormalities pointing toward periventricular leukomalacia, indicated by *red arrowheads. D*, MRI image of patient 2, showing no brain abnormalities.

Two male patients showed intellectual disability and a developmental delay with an unknown cause. Cognitive testing in patient 1, at the age of 5 years old, showed reduced intelligence (WPPSI-III-NL, score 2;9) and delay in adaptive behavior (VABS, score 2;0). Additional neurological symptoms were pyramidal syndrome, mild spastic diplegia, and psychomotor retardation with behavioral conduct disorder. In addition, patient 1 had an inguinal hernia. Dysmorphic features included hypertelorism, low-set ears, a broad nose, full lips, supernumerary nipple, hypoplastic toe, mild retrognathia, long and thin fingers, clinodactyly, and microcephaly (Fig. 1*A*). Patient 1 showed involvement of the eyes including amblyopia and possible astigmatism. He was born with a mild respiratory insufficiency based upon transient tachypnea of the neonate. Cardiac screening revealed a bicuspid aortic valve. An MRI at the age of 2 years old showed mild abnormalities pointing toward periventricular leukomalacia (Fig. 1*C*), but with a normal EEG.

The global developmental level of patient 2, at the age of 5.5 years old, was estimated at 1.5 years. He showed mouth hypotonia with drooling and involvement of the eyes including nystagmus, astigmatism, and high hypermetropia. Genital abnormalities included a small phallus, and orchiopexy was performed at the age of 3 years. His dysmorphic phenotype was less evident, with bitemporal narrowing and metopic ridge as main features (Fig. 1*B*). Upon birth, a ventricular septal defect was detected, which closed spontaneously at a later age. An MRI performed at the age of 3 years old showed no clear brain abnormalities (Fig. 1*D*). The clinical features of the patients are summarized in Table 1.

**Table 1:** **Comparison of clinical features of patients with hemizygote *OGT* mutations**

	Patient 1	Patient 2	Niranjan *et al.* (30)	Bouazzi *et al.* (29)
**Genotype**				
Genomic change (ChrX, GRCh38)	71,555,312G>C	71,544,561T>G	71,555,223G>T	71,555,984G>A
cDNA (NM_181672.2)	851 G>C	463–466 T>G	762 G>T	955 G>A
Protein	R284P		L254F	A319T

**Phenotype**				
Neurological features				
Intellectual disability	+	+	+	+
Developmental delay	+	+	ND*^a^*	+
Psychomotor retardation	+	+	ND	+
Behavioral problems	+	−	ND	+
Brain abnormalities	+	−	ND	−
Cardiac symptoms	+	+	ND	−
Dysmorphic features	+	+	ND	+
Eye abnormalities	+	+	ND	+
Genital/reproductive abnormalities	+	+	ND	−

*^a^* ND, not determined.

### Identification of mutations in OGT

Whole exome sequencing was performed in the two male patients from unrelated families to uncover the cause of their intellectual disability. Mutations in *OGT* were found in both patients.

**Figure 2.**
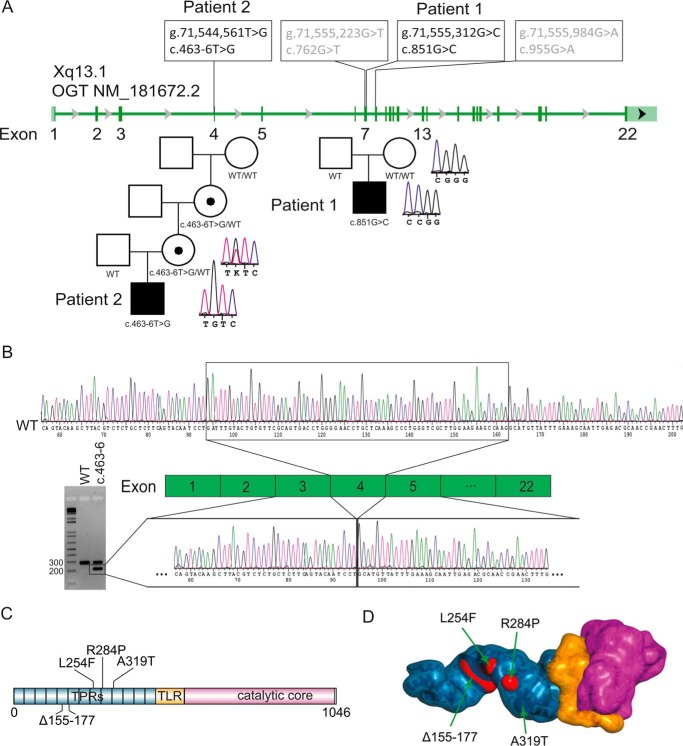
**Genetic analysis of hemizygous OGT mutations detected in patients with intellectual disability syndrome.** *A*, location of known and newly discovered genetic variants in the *OGT* gene and their presence in families 1 and 2. *B*, RT-PCR in skin-derived fibroblasts from patient 2 bearing the c.463–6T>G mutation confirmed by sequence analysis. *C*, domain organization in human *O*-GlcNAc transferase. *TLR*, tetratricopeptide-like repeat. *D*, model for the full-length human *O*-GlcNAc transferase generated by superposition of crystallographic models for the human *O*-GlcNAc transferase catalytic domain (Protein Data Bank code 5V1D) and tetratricopeptide repeat domain (Protein Data Bank code 1W3B). *Blue* represents tetratricopeptide repeats, *yellow* represents tetratricopeptide-like repeat, and *pink* represents catalytic core.

In patient 1, trio-sequencing and filtering for *de novo* variants revealed two potential candidate genes. A heterozygous *de novo* mutation was found in bromodomain-containing protein 1 (*BRD1*): Chr22(GRCh38): g.49,823,630C>G; NM_001304808.1: c.688G>C (p.Val230Leu). In addition, a hemizygous *de novo* missense variant in OGT was discovered: ChrX(GRCh38): g.71,555,312G>C; NM_181672.2: c.851G>C (p.Arg284Pro) (Fig. 2*A*). Arg-284 is conserved among many vertebrates and some invertebrates such as *Drosophila* (supplemental Fig. S1). *In silico* analysis of p.Arg284Pro in OGT predicted pathogenicity of the mutation (Table 2). The heterozygous nature of the mutation in *BRD1*, in addition to lower pathogenicity predictions for p.Val230Leu (Table 2), made this variant less likely to be pathogenic.

**Table 2:** **Prediction of pathogenicity of *de novo* mutations detected in patient 1**

	BRD1 p.Val230Leu	OGT p.Arg284Pro
Align GVGD	Class C0	Class C35
SIFT	Tolerated (score 0.06)	Deleterious (score 0.01)
MutationTaster	Disease causing (*p* value: 1)	Disease causing (*p* value: 1)
Polyphen-2	Possibly damaging (score: 0.544)	Probably damaging (score: 0.961)

For patient 2, a gene panel for known intellectual disability genes and *de novo* analysis did not reveal a potential causative mutation. Further analysis of the exome data by filtering for variants following an autosomal recessive or X-linked inheritance showed a hemizygous variant in *OGT*: ChrX(GRCh38): g.71,544,561T>G; NM_181672.2: c.463–6T>G. Segregation analysis revealed that the mutation originates from a *de novo* mutation in the grandmother of the patient, passed on via the mother, who is the carrier (Fig. 2*A*). The thymine at position c.463–6 is highly conserved (phyloP score of 6.93), and *in silico* splicing analysis of c.463–6T>G predicted an effect on the canonical splice site. RT-PCR in patient fibroblasts showed an additional splice variant for OGT (Fig. 2*B*). Sanger sequencing showed a deletion of 69 base pairs, corresponding to skipping of exon 4 caused by partial loss of the canonical splice site in patient 2 (Fig. 2*B*). Skipping of exon 4 could result in a truncated protein with a loss of 23 amino acids (Δ155–177-OGT).

Both mutations in OGT were absent in >60,000 controls (ExAC database) (31). Thus, we have identified two XLID patients that possess two novel mutations in *OGT.*

### OGT XLID mutations lead to an imbalance of O-GlcNAc homeostasis

**Figure 3.**
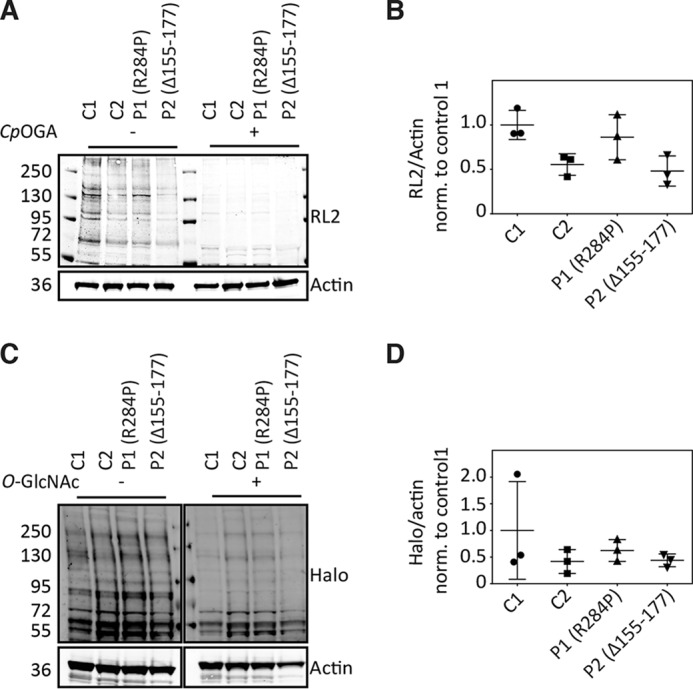
**Characterization of global *O*-GlcNAc levels in patient-derived skin fibroblasts.** *A*, immunoblot showing global *O*-GlcNAc levels in patient-derived fibroblasts detected using RL2 anti *O*-GlcNAc antibody. *C*, control; *P*, patient. *B*, the scatter plot indicates fractional ratio of any given actin-normalized signal averaged from three biological replicates, with the *error bars* representing the standard deviation. *p* values (Mann–Whitney *U* test): *C1–P1* = 0.4; *C1–P2* = 0.1; *C2–P1* = 0.4; *C2–P2* = 0.9. Achieved power (1 − β error probability) = 0.1. *C*, immunoblot showing global *O*-GlcNAc levels in patient-derived fibroblasts detected using the far Western method. The far Western method relies on detection of *O*-GlcNAcylated protein using a point mutant of a Halo-tagged bacterial *O*-GlcNAcase homolog, *Cp*OGA^D298N^, as a probe (32). Halo antibody was used to detect the bands where the probe was localized. *D*, the scatter plot indicates the fractional ratio of any given actin-normalized signal averaged from three biological replicates, with the *error bars* representing the standard deviation. *p* values (Mann–Whitney *U* test): *C1–P1* = 0.9; *C1–P2* = 0.7; *C2–P1* = 0.7; *C2–P2* = 0.9. Achieved power (1 − β error probability) = 0.1.

Given that OGT is a glycosyltransferase, we next investigated the effects of the XLID mutations on global *O*-GlcNAc levels in lysates from patient-derived fibroblasts by immunoblotting. As controls, we used fibroblasts from an unrelated male neonate (control 1) and a female adult (control 2). Using the RL2 anti-*O*-GlcNAc antibody, we observed no substantial difference in global *O*-GlcNAc levels between the controls and patients (Fig. 3, *A* and *B*). To verify the specificity of this *O*-GlcNAc antibody, cell lysates were treated with a bacterial *O*-GlcNAcase from *Clostridium perfringens* (*Cp*OGA), leading to a loss of *O*-GlcNAc signal compared with untreated lysates (Fig. 3*A*). As an independent control experiment, we applied a recently developed method to detect *O*-GlcNAcylated proteins, which relies on detection using a tagged inactive bacterial *O*-GlcNAcase (D298N-*Cp*OGA) (32). In this experiment, no substantial differences were observed in global *O*-GlcNAc levels in fibroblasts derived from patients *versus* healthy controls (Fig. 3, *C* and *D*).

**Figure 4.**
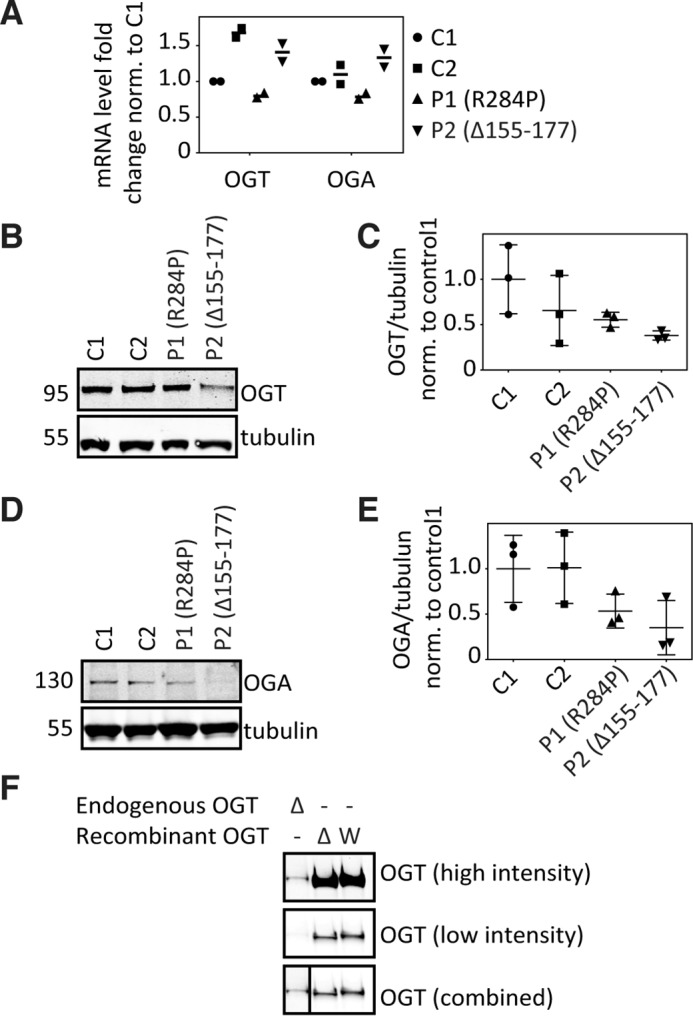
**Characterization of OGT and OGA levels in patient-derived skin fibroblasts.** *A*, gene expression of *OGT* and *OGA* in patient fibroblasts measured by qPCR. Scatter plots represent mean data from two independent experiments. Gene expression was calculated according to ΔΔ*C*(*t*) method. Tubulin expression was used as reference, and the data were normalized against control 1. *B*, immunoblot showing *O*-GlcNAc transferase (OGT) levels in patient-derived fibroblasts. *C*, control; *P*, patient. *C*, the scatter plot indicates fractional ratio of any given tubulin-normalized signal averaged from three biological replicates, with the *error bars* representing the standard deviation. *p* values (Mann–Whitney *U* test): *C1–P1* = 0.9; *C1–P2* = 0.1; *C2–P1* = 0.9; *C2–P2* = 0.7. Achieved power (1 − β error probability) = 0.1. *D*, immunoblot showing *O*-GlcNAcase (OGA) levels in patient-derived fibroblasts. *E*, the scatter plot indicates fractional ratio of any given tubulin-normalized signal averaged from three biological replicates, with the *error bars* representing the standard deviation. *p* values (Mann–Whitney *U* test): *C1–P1* = 0.2; *C1–P2* = 0.2; *C2–P1* = 0.2; *C2–P2* = 0.2. Achieved power (1 − β error probability) = 0.1. *F*, immunoblot comparing OGT protein molecular weight, as present in fibroblasts of patient 2 (Δ155–177) and of recombinantly expressed wild-type OGT (*W*) and Δ155–177-OGT (Δ).

We next investigated the effects of the *OGT* mutations on levels of OGT and OGA mRNA and protein. Gene expression levels of *OGT* and *OGA* appeared to be unaffected in patient fibroblasts compared with those of the controls (Fig. 4*A*). Although OGT protein levels appeared reduced in both patients compared with the healthy controls (Fig. 4, *B* and *C*), there was considerable variability in OGT protein levels of the healthy controls (Fig. 4, *B* and *C*). At the mRNA level, two OGT variants (corresponding to WT and mis-spliced products) were present for patient 2 (Fig. 2*B*). However, at the protein level, comparative analysis with recombinantly expressed Δ155–177-OGT (Fig. 4*F*) did not show evidence for presence of a truncated OGT protein, suggesting that Δ155–177-OGT may be unstable/degraded in the context of these patient fibroblasts. There was a reduction in OGA protein levels, although again variability was observed in OGA protein levels of the healthy controls (Fig. 4, *D* and *E*). Thus, *OGT* mutations may lead to reduced functional OGT protein levels in patient-derived fibroblasts, which may be compensated for with down-regulation of OGA protein levels, thereby maintaining global *O*-GlcNAc levels.

### Recombinant mutant OGT is unstable and defective in HCF-1 processing

**Figure 5.**
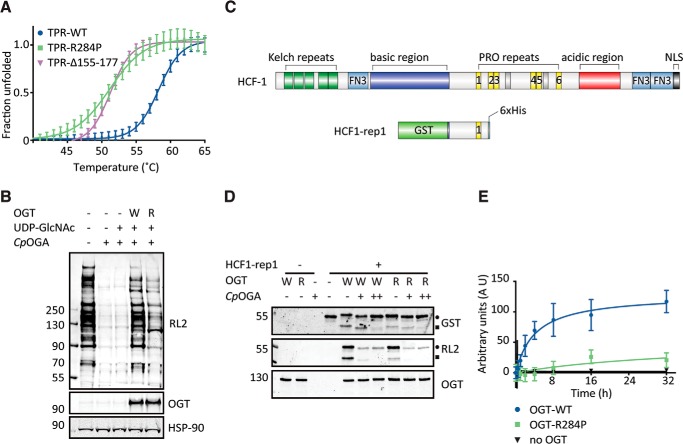
***In vitro* analysis of recombinantly expressed mutant OGT forms.** *A*, thermal denaturing curve showing fraction of unfolded wild-type (*TPR-WT*), R284P (*TPR-R284P*), and Δ155–177 (*TPR*-Δ*155–177*) OGT TPR domains as a function of time. The data were fitted to Boltzmann sigmoidal curve equation. *Error bars* represent standard error of mean of seven replicates, and the plot is representative of three biological replicates. *B*, immunoblot showing the relative *O*-GlcNAcylation activities of wild-type OGT (*W*) and R284P-OGT (R) against de-*O*-GlcNAcylated HEK-293 lysate. *C*, domain organization of human HCF1 compared with its truncated form fused to N-terminal GST tag and C-terminal His tag (HCF1-rep1), which was used as a substrate for OGT proteolytic activity. *D*, immunoblot showing the relative proteolytic activities of wild-type OGT (*W*) and R284P-OGT (*R*) against HCF1-rep1. The samples were treated with an *O*-GlcNAcase homolog from *C. perfringens* (*Cp*OGA) either toward the end of the reaction (+) or from the beginning (++). *Closed circle*, unprocessed HCF-1rep1; *closed square*, HCF1-rep1 cleavage product. *E*, kinetic assay of the proteolytic activity of wild-type OGT and R284P-OGT against HCF1-rep1 plotted as amount of product formed as a function of time. *Error bars* represent standard error of mean of four replicates. The data were fitted to hyperbolic curve equation.

The absence of the truncated OGT in patient 2 and potential reductions in OGT protein levels in patient-derived fibroblasts suggest that the mutations may compromise OGT stability. To further investigate this, stability of recombinant wild-type/mutant OGT TPR domain was analyzed by thermal denaturation experiments. We observed reduced stability for Δ155–177-OGT (*T*_m_ = 51 ± 1 °C) and R284P-OGT (*T*_m_ = 52 ± 1 °C) compared with wild-type OGT TPR domain (*T*_m_ = 59 ± 1 °C) (Fig. 5*A*). Given that Δ155–177-OGT was not detected in patient-derived fibroblasts, further biochemical characterization of this mutant was omitted. OGT has over 1000 *O*-GlcNAcylation substrates; therefore, to explore the effects of the p.Arg284Pro mutation in an environment similar to that of cell physiology, de-*O*-GlcNAcylated HEK-293 cell lysate was employed as a complex array of substrates. R284P-OGT was not able to restore protein *O*-GlcNAcylation, to the extent achieved by wild-type OGT (Fig. 5*B*).

OGT is known to possess a second catalytic activity, promoting autocatalytic cleavage and activation of the transcriptional co-regulator HCF1, itself an XLID gene (33, 34), via an intermediate glycosylation step (13, 35). Thus, we decided to investigate the effects of the p.Arg284Pro substitution on OGT-mediated HCF1 processing. To measure proteolytic activity, we used a GST fusion of a minimal HCF1 fragment (HCF1-rep1) possessing the first of the PRO repeats (which are the target sites of proteolysis; Fig. 5*C*). Wild-type OGT was able to hyperglycosylate HCF1-rep1, as evidenced by a band shift that was reversible by incubation with *Cp*OGA (Fig. 5*D*). The HCF1-rep1 band shift observed in the R284P-OGT-catalyzed reaction was smaller, suggesting that the mutant OGT was less effective than the wild type in glycosylating HCF1-rep1 (Fig. 5*D*). Furthermore, wild-type OGT was able to induce autoproteolysis, whereas the proteolytic activity of R284P-OGT was reduced (Fig. 5*D*). When the enzyme concentration was reduced relative to substrate levels, proteolytic activity of R284P-OGT was abrogated, whereas wild-type enzyme still displayed detectable activity (Fig. 5*E*). Thus, recombinant R284P-OGT is defective in HCF1 glycosylation and proteolytic processing.

## Discussion

Here, we report two patients with XLID attributable to hemizygous mutations in *OGT*: one missense mutation (p.Arg284Pro) and one splice-site mutation (c.463–6T>G). We identified potential perturbations in protein *O*-GlcNAc homeostasis in both patients and altered HCF1 maturation in patient 1.

XLID is a genetically and phenotypically heterogeneous group of disorders, estimated to account for ∼10% of all intellectual disability phenotypes in males (36). Mutations causing XLID have been reported in over 100 genes, and new XLID genes are still being identified (37). In 2015, Niranjan *et al.* (30) published a series of 65 potential XLID genes, by sequencing the X-exome of 56 XLID families. This was the first publication in which a genetic variant in *OGT* (p.Leu254Phe) was found associated with XLID. During the course of our work, Vaidyanathan *et al.* (38) reported a study on the functional characterization of this previously published p.Leu254Phe variant of OGT (30). In patient-derived lymphoblastoid cells, decreased OGT levels were accompanied by decreased OGA levels, resulting in unaltered *O*-GlcNAc levels. This is in agreement with our findings in both patient-derived fibroblast cell lines, indicating that homeostasis of *O*-GlcNAc levels through decreasing OGA could be a general principle in cases of OGT deficiency. However, in contrast to our results, recombinantly expressed p.Leu254Phe OGT and wild-type OGT showed equal activity toward *O*-GlcNAc substrate CK2α and HCF-1 (38). Another study described a missense mutation in *OGT* (p.Ala319Thr) in an XLID family, although this mutant also segregated with a previously uncharacterized variant of *MED12*, another gene that is associated with XLID. Evidence for functional loss of OGT for this mutation was not provided (29).

To date, including our findings, four hemizygous mutations in *OGT* have been identified in patients with XLID (Table 1). The three missense mutations (p.Arg284Pro, p.Leu254Phe, and p.Ala319Thr) and one splice-site mutation (c.463–6T>G) all affect the N-terminal TPR domain (Fig. 2*C* and supplemental Fig. S1). The TPR motif is characterized by a repeat of 34 amino acids that fold into two antiparallel helices. The TPR domain consists of 13.5 repeats, and the antiparallel helices align to form an elongated superhelix, where the first helix of each TPR repeat (helix A) faces toward the inner surface of the elongated superhelix and their antiparallel pairs (helix B) form the outer surface (11). The missense mutations p.Arg284Pro, p.Leu254Phe, and p.Ala319Thr are located in helix B of the OGT TPR7, TPR8, and TPR9, respectively (Fig. 2*D* and supplemental Fig. S1). In addition, Ala-319 is one of the conserved hydrophobic amino acid residues of the canonical TPR repeat (39) (supplemental Fig. S1). Each of these missense mutations could disrupt the α-helical secondary structure of the TPR domain, thereby affecting the overall stability of the elongated superhelix (Fig. 2*D*). Arg-284 is located in the middle of TPR helix 7B, and mutation of this residue to a Pro likely disrupts this secondary structure element, destabilizing this region.

The splice-site mutation c.463–6T>G is predicted to result in an OGT protein missing 23 amino acids (p.Δ155–177) (Fig. 2*B*), although we could not confirm the presence of a truncated OGT in patient fibroblasts (Fig. 4*F*). We propose that this mutation might exert its effect by producing an unstable splice variant and reducing the amount of functional OGT within the cell (Figs. 4*B* and 5*A*). Global *O*-GlcNAc levels in patient-derived fibroblasts were not substantially reduced compared with healthy controls (Fig. 3). Protein levels of OGT and OGA appeared to be reduced in both patients; however, both healthy controls displayed variation in their OGT and OGA levels, complicating the interpretation of these findings. We hypothesize that the decrease in OGT protein levels in these cells might be counteracted by the concomitant reduction of OGA protein levels (Fig. 4, *D* and *E*), thereby sustaining basal *O*-GlcNAc homeostasis. This hypothesis is supported by the finding that chemical inhibition of OGA in different human cell lines leads to a decreased expression of OGT and an increased expression of OGA (18), which indicates an as-yet-unidentified mechanism of regulation to maintain global *O*-GlcNAc homeostasis. We propose a similar mechanism in our patient-derived fibroblasts.

It is as yet not fully understood how OGT recognizes serines/threonines on specific proteins for *O*-GlcNAcylation, although there is evidence to suggest that the TPRs play a key role for at least a subset of substrates (40). To assess the *in vitro* activity of OGT with mutations affecting the TPR domain and the impact on substrate *O*-GlcNAcylation, we produced recombinant R284P-OGT. Given that Δ155–177-OGT was not detected in patient-derived fibroblasts, further biochemical characterization of this mutant was not undertaken. Activity assays against de-*O*-GlcNAcylated HEK-293 lysate demonstrated that the p.Arg284Pro mutation led to a global reduction in protein *O*-GlcNAcylation, as opposed to reducing recognition and *O*-GlcNAcylation of specific substrates. In a physiological context, this nonspecific reduction in OGT activity may be rescued by down-regulation of OGA levels, which was observed to some extent in the fibroblasts from both patients. However, the p.Arg284Pro mutation is located on the surface of the TPRs (11), a region that has been shown to play a role in the recognition of specific substrates (40). It is therefore possible that this mutation could affect modification of specific substrates, in addition to causing the observed global reduction in substrate *O*-GlcNAcylation, and this possibility is neither excluded nor confirmed by our current data. *O*-GlcNAcylation activity of R284P-OGT against HCF1-rep1 was reduced, as judged by the poly-*O*-GlcNAcylation-induced shift in HCF1-rep1 molecular weight *versus* wild-type OGT (Fig. 5*D*).

In addition to being abundantly *O*-GlcNAcylated (15), HCF1 is also proteolytically processed by OGT (12, 35). The p.Arg284Pro mutation appears to alter HCF1-rep1 proteolysis *in vitro* (Fig. 5, *D* and *E*). The TPR domain is critical for proteolytic maturation of HCF1 (13), and this could explain why R284P-OGT shows decreased proteolytic activity toward HCF1 *in vitro*. Interestingly, *HCFC1*, the gene that codes for HCF1, is located on the X-chromosome, and it is reported as an XLID gene (41). This hints at possible overlap in downstream disease mechanisms causing intellectual disability in patients bearing *OGT* or *HCFC1* mutations.

Precisely how alterations in protein *O*-GlcNAcylation and HCF1 cleavage lead to intellectual disability is not yet clear. OGT is expressed in a broad range of tissues, with a remarkably high expression in brain (9, 10). *O*-GlcNAc-modified proteins were detected in nerve terminals, and abundant OGT activity was detected in nerve synaptosomes (42), indicating that *O*-GlcNAc could play a role in neuronal function and neurodegenerative disease. It has been suggested that regulation of the proteasome complex by *O*-GlcNAcylation in the brain could contribute to apoptosis of hippocampal cells and thus neurodegeneration (43). In addition, aberrant *O*-GlcNAcylation levels have been linked to neurodegenerative diseases like Alzheimer's disease (28). Future work analyzing the effect of XLID mutations on OGT function in a more phenotype-relevant model, such as a neuronal cell line or an animal model where the effect of the mutations on different tissues, including the brain, can be investigated, would be important. The present study utilized fibroblasts from two unrelated individuals as controls, and we noted substantial variability in OGA and OGT protein levels in these cells, complicating the interpretation of our data. To minimize the effect of variability introduced by the differing genetic background between individual patients and controls, it would be of use to introduce these mutations into cell lines with an identical genetic background. Our study provides the first detailed clinical characterization of patients with XLID attributable to mutations in OGT and furthermore proposes a potential molecular explanation for how these mutations may impact on OGT dual function of *O*-GlcNAcylation and HCF1 proteolysis.

## Experimental procedures

### Patient consent

The study was approved by the Institutional Review Board at Radboud University Medical Centre and Utrecht University Medical Centre. All participating affected individuals or their legal representatives gave informed consent for exome sequencing and to publication of this study. Fibroblasts of the patients were obtained for diagnostics of inborn errors of metabolism and used after informed consent from parents and/or treating physicians, in accordance with the Declaration of Helsinki. For use of facial images, written informed consent was obtained.

### Whole exome sequencing

For patient 1, whole exome sequencing was performed in a trio diagnostic approach as described before (44). Exome capture was performed with the SureSelect Human All Exon v4 enrichment kit (Agilent, Santa Clara, CA). Whole exome sequencing was performed on the Illumina HiSeq platform (San Diego, CA). Data were analyzed with the BWA (read alignment) and GATK (variant calling) software packages. Variants were annotated using an in-house developed pipeline. Prioritization of variants was done by an in-house designed “variant interface” and manual curation.

Exomes of patient 2 and parents were enriched using the SureSelect XT Human All Exon V5 kit (Agilent) and sequenced in rapid run mode on the HiSeq2500 sequencing system (Illumina) at a mean target depth of 100×. The target is defined as all coding exons of UCSC and Ensembl ± 20-bp intron flanks. At this depth >95% of the target is covered at least 15 times. Reads were aligned to hg19 using BWA (BWA-MEM v0.7.5a), and variants were called using the GATK haplotype caller (v2.7-2). Detected variants were annotated, filtered, and prioritized using the Bench NGS Lab platform (Cartagenia; Agilent). The *OGT* gene sequences of the grandmother and great-grandmother were analyzed to study the origin of the mutation. All potentially causative variants were confirmed by Sanger sequencing.

### Cell culture

Skin fibroblasts derived from patients and healthy donors were cultured at 37 °C under 5.0% CO_2_ in M199 medium (PAN Biotech, Aidenbach, Germany) or Dulbecco's modified Eagle's medium (Thermo Fisher Scientific) containing 2 mm l-glutamine (Sigma). Media were supplemented with 10% fetal calf serum (Labtech, Uckfield, UK) and 1% penicillin-streptomycin (Thermo Fisher Scientific).

### RNA extraction, cDNA synthesis, and quantitative RT-PCR

Total cell RNA was extracted from skin fibroblasts using TRIzol reagent (Thermo Fisher Scientific). cDNA was synthesized with a RevertAid First Strand cDNA synthesis kit (Thermo Fisher Scientific) using random hexamer primers. For quantification of OGT and OGA expression levels, cDNA from skin fibroblasts was mixed with GoTaq® qPCR Master Mix (Promega) and both forward and reverse primers. Tubulin expression was used as an internal control. All qPCR experiments were conducted at Bio-Rad CFX96 real-time system following the GoTaq® qPCR Master Mix manual. Specificity of the reactions was verified with melt curve analysis. The primers used are listed in supplemental Table S1. For analysis of OGT mRNA splicing in skin fibroblaststk;1 of patient 2, cDNA and primers were mixed with AmpliTaq Gold® 360 Master Mix (Thermo Fisher Scientific). RT-PCR was conducted according to standard conditions and PCR products were purified using a QIAquick gel extraction kit (Qiagen). Purified PCR products were used for sequencing analysis.

### Biochemical characterization of patient-derived fibroblasts

Patient-derived fibroblasts were grown on 15-cm plates and were washed twice with ice-cold PBS buffer (Thermo Fisher Scientific) prior to lysis. The cells were lysed by addition of lysis buffer (50 mm Tris-HCl, pH 7.4, 1 mm EGTA, 1 mm EDTA, 1% Triton X-100, 1 mm Na_3_VO_4_, 50 mm NaF, 5 mm pyrophosphate, 0.27 m sucrose) supplemented with 1 μm GlcNAcstatin-G, 1 μm β-mercaptoethanol, and protease inhibitor mixture (1 mm benzamidine, 0.2 mm PMSF, 5 mm leupeptin). The lysate was transferred into an Eppendorf and clarified by centrifugation at 4 °C (1200 × *g* for 15 min). Lysate proteins were resolved by SDS-PAGE (4–12% acrylamide; Thermo Fisher Scientific) and transferred onto nitrocellulose membranes (GE Healthcare). Analysis of *O*-GlcNAcylated fibroblast proteins by the far Western method was performed as described previously (32). Briefly, soluble cell lysates were prepared, resolved by SDS-PAGE (3–8% Tris acetate; Thermo Fisher Scientific) and transferred onto nitrocellulose membranes (GE Healthcare) as described above, but with lysis buffer lacking GlcNAcstatin-G.

### Protein expression and purification

Full-length wild-type OGT and mutants (R284P and Δ155–177) were expressed in *Escherichia coli* as N-terminal His fusion proteins as described previously (45). The wild-type OGT TPR domain (residues 26–420) and mutants (TPR-R284P and TPR-Δ155–177) were expressed and purified as described previously (11). HCF1-rep1 (residues 867–1071) was expressed in *E. coli* as a non-cleavable N-terminal GST and C-terminal His fusion protein. Transformed BL21 (DE3) cells were grown, induced for expression, lysed, and clarified as described previously (45). Clarified lysate was incubated with 1 ml/liter of culture of glutathione-Sepharose 4B resin (GE Healthcare) for 2 h at 4 °C. The resin was thoroughly washed with base buffer (0.1 m Tris-HCl, pH 7.5, 150 mm NaCl, 0.5 mm TCEP) and eluted using base buffer supplemented with 0.5 m glutathione. Eluted protein was dialyzed overnight at 4 °C in buffer A (0.1 m Tris-HCl, pH 7.5, 25 mm NaCl). Dialyzed protein was passed through 5 ml of HiTrap Q-Sepharose FF anion exchange resin (GE Healthcare), collecting the flow-through, which was then concentrated and passed through a 300-ml Superdex^TM^ 200 column (GE Healthcare) pre-equilibrated with base buffer. The peak fractions were concentrated to 10 mg/ml, mixed 1:1 with 50% glycerol, snap-frozen, and stored at −80 °C until use.

### Molecular cloning

The fragment of HCF1-rep1 (residues 867–1071) was obtained from MVP human total brain RNA from Agilent using the Takara PrimeScript high-fidelity RT-PCR kit. The reverse primer coded for the C-terminal addition of a His_6_ tag. The PCR product was cloned as a BamHI-NotI fragment into a mutated version of pGEX6P1, which lacks the PreScission protease site. The full-length codon optimized OGT was obtained from GenScript and subcloned as a BamHI-NotI fragment into pHEX6P1 (modified version of pGEX6P1 which contains a His_6_ tag instead of GST). The R284P and the Δ155–177 mutations were introduced using a method similar to the QuikChange site-directed mutagenesis kit (Agilent) but using KOD polymerase and DpnI from Fermentas. All inserts were confirmed by DNA sequencing.

### Thermal denaturing assay

Thermal denaturing experiments were performed in triplicate, using the OGT TPR domain constructs. 50-μl solutions contained 5 μm protein and 1.1x SYPRO® Orange dye (Sigma) in base buffer of 25 mm HEPES-NaOH, pH 7.5, 150 mm NaCl, and 0.5 mm TCEP. A Bio-Rad (CFX Connect^TM^ real-time system was used to measure fluorescence (λ_ex_ = 530 nm, λ_em_ = 560 nm) while temperature was increased from 25 to 95 °C at 1°/min increments. The data were transformed, normalized, and fitted to a Boltzmann sigmoidal curve using GraphPad Prism 5.0.

### In vitro O-GlcNAcylation assay

*O*-GlcNAcylation assays were performed in triplicate on de-*O*-GlcNAcylated HEK-293 lysate proteins. HEK-293 cell lysates were prepared as described for patient-derived fibroblasts, but with the lysis buffer lacking GlcNAcstatin-G. HEK-293 lysate was treated with 120 μg of *Cp*OGA per mg of lysate protein and incubated for 90 min at 37 °C. *Cp*OGA was neutralized by the addition of 250 μm GlcNAcstatin-G. Reactions were supplemented with wild-type OGT or R284P-OGT (0.2 μm) in the presence of 2 mm UDP-GlcNAc and incubated for an additional 2 h at 37 °C. Proteins were resolved by SDS-PAGE (3–8% acrylamide; Thermo Fisher Scientific) and transferred onto nitrocellulose membrane (GE Healthcare).

### In vitro proteolytic assay

Proteolytic assays were performed using HCF1-rep1 (residues 867–1071) with GST and His tags at the N and C termini, respectively. For qualitative measurement of proteolytic activity, HCF1-rep1 (2.5 μm), preincubated or not with *Cp*OGA (5 μm), was combined with wild-type OGT or R284P-OGT (1 μm) in the presence of 1 mm UDP-GlcNAc. Reaction mixtures were incubated at 37 °C for 8 h with gentle agitation, followed by optional addition of *Cp*OGA. Mixtures were incubated at 37 °C for an additional 1 h, before stopping the reaction by addition of LDS loading buffer (4×) (Thermo Fisher Scientific). Proteins were resolved by SDS-PAGE (4–12% acrylamide; Life Technologies) and transferred onto nitrocellulose membranes (GE Healthcare). Initial velocity for OGT-catalyzed proteolysis of HCF1-rep1 was measured in triplicate. HCF1-rep1 (1.5 μm) was combined with wild-type OGT or R284P-OGT (0.15 μm) in the presence of 150 μm UDP-GlcNAc. Reaction mixtures were incubated at 37 °C with gentle agitation, whereas aliquots were taken out at the designated time points (0, 0.5, 1, 2, 4, 8, 16, and 32 h) and stopped by mixing with LDS loading buffer (4×) (Thermo Fisher Scientific). The initial volume of the reaction was 90 μl, and 2.5-μl aliquots were loaded in each lane. Proteins were resolved by SDS-PAGE (10% acrylamide) and visualized by Coomassie staining. The amount of product formed was quantified using a LI-COR Odyssey scanner and associated quantification software. The data were background-corrected and plotted using GraphPad Prism 5.0.

### Immunoblots

Membranes were probed with following primary antibodies: *O*-GlcNAc (RL2; Abcam, Cambridge, UK), actin (Sigma), OGT (H300; Santa Cruz), OGA (14711-1-AP; Proteintech, Rosemont, IL), tubulin (Cell Signaling), HSP-90 (Cell Signaling), and GST-S902 (Division of Signal Transduction Therapy, University of Dundee). In case of the far Western method, membranes were probed with 10 μg/ml Halo-tagged *Cp*OGA and incubated with halo primary antibody (Promega). Subsequently all membranes were incubated with IR680- or IR800-labeled secondary antibodies (LI-COR) and analyzed using a LI-COR Odyssey scanner and associated quantification software. All primary and secondary antibodies were used in 1:5,000 and 1:10,000 dilutions, respectively.

### Data analysis

Scatter plots indicate ratios of any given normalized signal as averaged from three biological replicates, with the *error bars* representing standard deviation. Variations in data were calculated by Mann–Whitney *U* test using GraphPad Prism 5.0. Power calculations were performed using G*Power 3.1.

## Author contributions

A. P. W. and M. G. designed, performed, and analyzed the experiments and wrote the paper. M. J. E. K., J. C. G., B. L., and J. F. provided clinical data of the patients. R. P., M. E., and K. L. I. V. G. performed exome sequencing and provided genetic details of the patients. A. T. F. performed molecular cloning to obtain recombinantly expressed OGT forms. D. J. L. and D. M. F. V. A. conceived and coordinated the study and revised the paper. All authors contributed to writing of the paper, reviewed the results, and approved the final version of the manuscript.

## Supplementary Material

Supplemental Data

## Footnotes

5 The abbreviations used are:

*O*-GlcNAc: *N*-acetylglucosamine
XLID: X-linked intellectual disability
OGA: *O*-GlcNAcase
OGT: *O*-GlcNAc transferase
HCF1: host cell factor 1
TPR: tetratricopeptide repeat
qPCR: quantitative PCR.
