# Supplementary material for: Mutations in N-acetylglucosamine (O-GlcNAc) transferase in patients with X-linked intellectual disability
Source: J Biol Chem. 2017 Jun 5;292(30):12621–31. doi: 10.1074/jbc.M117.790097 (PMC5535036; doi:10.1074/jbc.M117.790097)
Supplement: Supplemental Data [file supp_292_30_12621__index.html]

Mutations in N-acetylglucosamine (O-GlcNAc) transferase in patients with X-linked intellectual disability — Mutations in N-acetylglucosamine (O-GlcNAc) transferase in patients with X-linked intellectual disability — OGT variants in XLID — Supplemental Data 

# Mutations in *N*-acetylglucosamine (*O*-GlcNAc) transferase in patients with X-linked intellectual disability

## Supplemental Data

- Supplemental material (.pdf, 633 KB) - This file contains 1 supplemental figure and 1 supplemental table
