# Supplementary material for: Mutations in N-acetylglucosamine (O-GlcNAc) transferase in patients with X-linked intellectual disability
Source: J Biol Chem. 2017 Jun 5;292(30):12621–31. doi: 10.1074/jbc.M117.790097 (PMC5535036; doi:10.1074/jbc.M117.790097)
Supplement: Supplemental Data [file 10.1074_M117.790097_jbc.M117.790097-1.pdf]

Mutations in *N*-acetylglucosamine (O-GlcNAc) transferase in patients with X-linked intellectual disability

Anke P. Willems, Mehmet Gundogdu, Marlies J.E. Kempers, Jacques C. Giltay, Rolph Pfundt, Martin Elferink, Bettina F. Loza, Joris Fuijkschot, Andrew T. Ferenbach, Koen L.I. van Gassen, Daan M.F. van Aalten and Dirk J. Lefeber

This supplemental material section includes:

Supplemental Figure S1
Supplemental Table S1

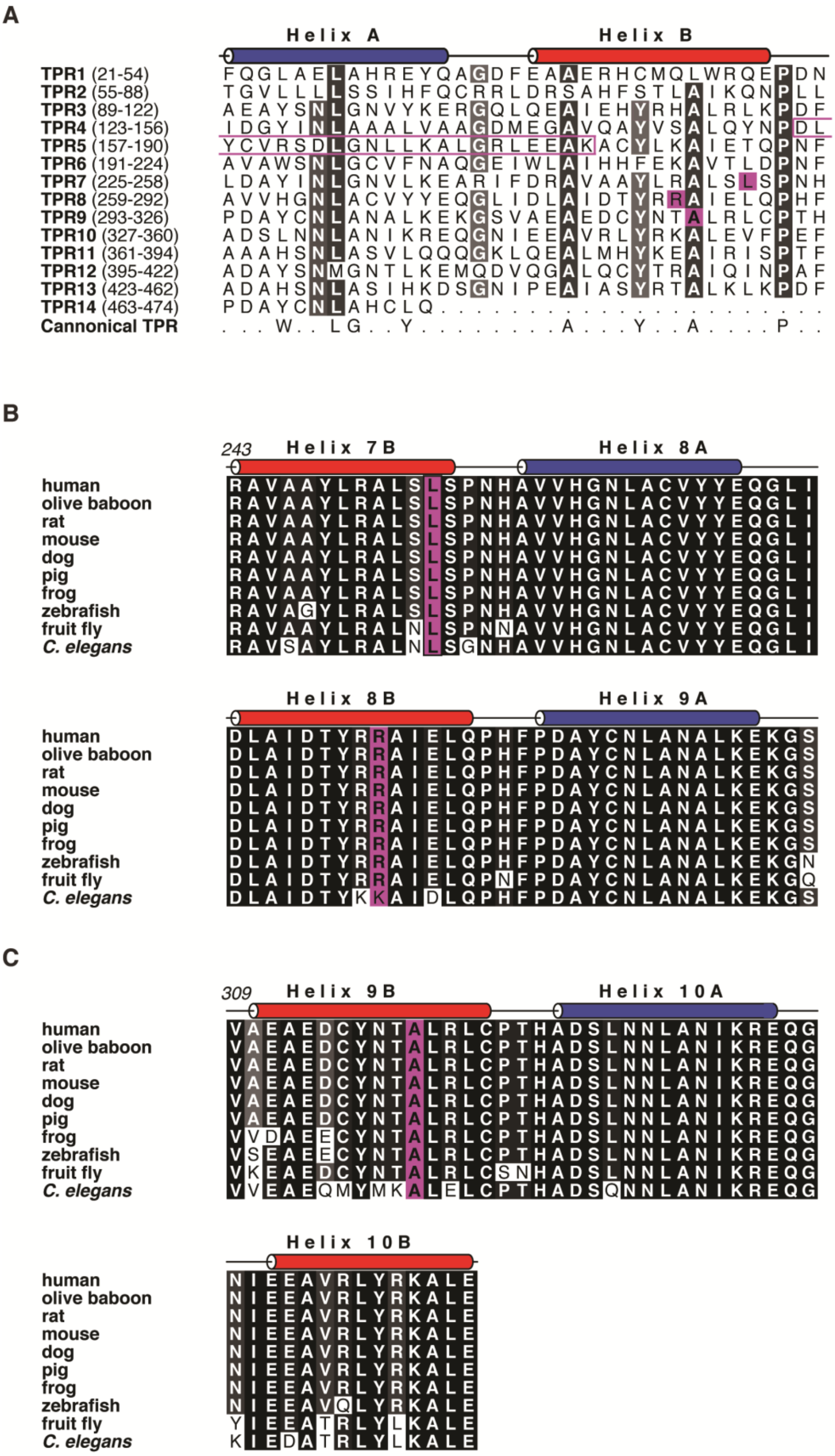

A
Helix A
Helix B
TPR1 (21-54) FQGLAELAHREYQAGDFEAAERHCMQLWRQEPDN
TPR2 (55-88) TGVLLLLSSIHFQCRRLDRSAHFSTLAIKQNPLL
TPR3 (89-122) AEAYSNLGNVYKERGQLQEAIEHYRHALRLKPDF
TPR4 (123-156) IDGYINLAAALVAAGDMEGAVQAYVSALQYNPDL
TPR5 (157-190) YCVRSDLGNLLKALGRLEEAKACYLKAIETQPNF
TPR6 (191-224) AVAWSNLGCVFNAQGEIWLAIHHFEKAVTLDPNF
TPR7 (225-258) LDAYINLGNVLKEARIFDRAVAAYLRALSLSPNH
TPR8 (259-292) AVVHGNLACVYYEQGLIDLAIDTYRRAIELQPHF
TPR9 (293-326) PDAYCNLANALKEKGSVAEAEDCYNTALRLCPTH
TPR10 (327-360) ADSLNNLANIKREQGNIEEAVRLYRKALEVFPEF
TPR11 (361-394) AAAHSNLASVLQQQGKLQEALMHYKEAIRISPTF
TPR12 (395-422) ADAYSNMGNTLKEMQDVQGALQCYTRAIQINPAF
TPR13 (423-462) ADAHSNLASIHKDSGNIPEAIASYRTALKLKPDF
TPR14 (463-474) PDAYCNLAHCLQ......................
Cannonical TPR ...W..LG..Y........A...Y..A....P..
B
243
Helix 7B
Helix 8A
human RAVAAYLRALSLSPNHAVVHGNLACVYYEQGLI
olive baboon RAVAAYLRALSLSPNHAVVHGNLACVYYEQGLI
rat RAVAAYLRALSLSPNHAVVHGNLACVYYEQGLI
mouse RAVAAYLRALSLSPNHAVVHGNLACVYYEQGLI
dog RAVAAYLRALSLSPNHAVVHGNLACVYYEQGLI
pig RAVAAYLRALSLSPNHAVVHGNLACVYYEQGLI
frog RAVAAYLRALSLSPNHAVVHGNLACVYYEQGLI
zebrafish RAVAGYLRALSLSPNHAVVHGNLACVYYEQGLI
fruit fly RAVAAYLRALNLSPNNAVVHGNLACVYYEQGLI
C. elegans RAVSAYLRALNLSGNHAVVHGNLACVYYEQGLI
Helix 8B
Helix 9A
human DLAIDTYRRAIELQPHFPDAYCNLANALKEKGS
olive baboon DLAIDTYRRAIELQPHFPDAYCNLANALKEKGS
rat DLAIDTYRRAIELQPHFPDAYCNLANALKEKGS
mouse DLAIDTYRRAIELQPHFPDAYCNLANALKEKGS
dog DLAIDTYRRAIELQPHFPDAYCNLANALKEKGS
pig DLAIDTYRRAIELQPHFPDAYCNLANALKEKGS
frog DLAIDTYRRAIELQPHFPDAYCNLANALKEKGS
zebrafish DLAIDTYRRAIELQPHFPDAYCNLANALKEKGN
fruit fly DLAIDTYRRAIELQPNFPDAYCNLANALKEKGQ
C. elegans DLAIDTYKKAIDLQPHFPDAYCNLANALKEKGS
C
309
Helix 9B
Helix 10A
human VAEAEDCYNTALRLCPTHADSLNNLANIKREQG
olive baboon VAEAEDCYNTALRLCPTHADSLNNLANIKREQG
rat VAEAEDCYNTALRLCPTHADSLNNLANIKREQG
mouse VAEAEDCYNTALRLCPTHADSLNNLANIKREQG
dog VAEAEDCYNTALRLCPTHADSLNNLANIKREQG
pig VAEAEDCYNTALRLCPTHADSLNNLANIKREQG
frog VVDAEECYNTALRLCPTHADSLNNLANIKREQG
zebrafish VSEAEECYNTALRLCPTHADSLNNLANIKREQG
fruit fly VKEAEDCYNTALRLCSNHADSLNNLANIKREQG
C. elegans VVEAEQMYMKALELCPTHADSQNNLANIKREQG
Helix 10B
human NIEEAVRLYRKALE
olive baboon NIEEAVRLYRKALE
rat NIEEAVRLYRKALE
mouse NIEEAVRLYRKALE
dog NIEEAVRLYRKALE
pig NIEEAVRLYRKALE
frog NIEEAVRLYRKALE
zebrafish NIEEAVQLYRKALE
fruit fly YIEEATRLYLKALE
C. elegans KIEDATRLYLKALE

Fig. S1. Sequence alignment of TPRs

A. Sequence alignment of the 13.5 human OGT TPRs. Blue and red cylinders annotate the first helix (helix A) and its antiparallel pair (helix B) within each TPR motif, respectively. The alignment is coloured in grayscale based on sequence conservation. The regions affected by all four XLID mutations, Δ155-177, Leu254Phe, Arg284Pro and Ala319Thr, are highlighted in pink colour.

B. Sequence alignment of OGT exon 7 from various metazoan species. Blue and red cylinders annotate the first helix (helix A) and its antiparallel pair (helix B) within each TPR motif, respectively. The alignment is coloured in grayscale based on sequence conservation. The XLID mutations Leu254Phe and Arg284Pro are highlighted in pink colour.

C. Sequence alignment of OGT exon 8 from various metazoan species. Blue and red cylinders annotate the first helix (helix A) and its antiparallel pair (helix B) within each TPR motif, respectively. The alignment is coloured in grayscale based on sequence conservation. The XLID mutation Ala319Thr is highlighted in pink colour.

Table S1: Overview of used primers

**Analysis of mRNA expression by qPCR**	
OGT (Fw)	5'- GCCAGCTCCTATTCAGGCAA -3'
OGT (Rv)	5'- CCATGAAAAGCGCACCACTC -3'
OGA (Fw)	5'- TGCGATCTCACCTGGATTGG -3'
OGA (Rv)	5'- CCAAACTGAGAAACCTGGTCC -3'
Tubulin (Fw)	5'- CAATGAAGCCACAGGTGGCAAA -3'
Tubulin (Rv)	5'- GTTGTTACCTGCCCCAGACT-3'
**Analysis of OGT mRNA splicing**	
Fw	5'- GCATTGCGTCTCAAACCTGA-3'
Rv	5'- AAGCCAAATTTCCCCTTGTGC-3'
**Molecular cloning**	
hOGT_R284P_fwd	GCTATTGATACCTATCGTCcgGCGATTGAACTGCAGCCG
hOGT_R284P_rev	CGGCTGCAGTTCAATCGCcgGACGATAGGTATCAATAGC
hOGT_delta_155_177_fwd	gtctctgcgctgcagtataacccggcctgttacctgaaagcgattgaaacc
hOGT_delta_155_177_rev	ggtttcaatcgctttcagg taacaggccgggttatactgcagcgcagagac
HCF_fwd	aaaGGATCCccagccgtcaccacgttggttgtg
HCF_rev	aaaGCGGCCGCttaGTGGTGATGATGGTGATGcacgctaccattctgctggcccac
